# Draft genome sequence of the basidiomycetous yeast *Mrakia hoshinonis* JCM 32575^T^ isolated from Ellesmere Island, Canadian High Arctic

**DOI:** 10.1128/mra.00820-23

**Published:** 2024-01-05

**Authors:** Tatsuya Fujikawa, Jun-ichi Ishihara, Warwick F. Vincent, Masaki Uchida, Masaharu Tsuji

**Affiliations:** 1 Department of Materials Chemistry, National Institute of Technology (KOSEN), Asahikawa College, Asahikawa, Hokkaido, Japan; 2 Medical Mycology Research Center, Chiba University, Chiba, Japan; 3 Département de Biologie & Centre for Northern Studies (CEN), Université Laval, Quebec, Canada; 4 National Institute of Polar Research (NIPR), Tachikawa, Tokyo, Japan; 5 Department of Polar Science, The Graduate University for Advanced Studies, Tachikawa, Tokyo, Japan; University of Maryland School of Medicine, USA

**Keywords:** high Arctic, draft genome sequence, yeasts, cold-adapted

## Abstract

*Mrakia hoshinonis* JCM 32575 was isolated from glacial sediments on Ellesmere Island in the Canadian High Arctic and described as a new basidiomycetous yeast. This species does not require amino acids and vitamins for growth and can grow at sub-zero temperatures. Here, we report a draft genome sequence of this strain.

## ANNOUNCEMENT

*Mrakia hoshinonis* was isolated as a new basidiomycetous yeast species from sediments at the front of a retreating glacier on northern Ellesmere Island in the Canadian High Arctic (1), an important conservation site in the coastal margin of the Last Ice Area (2). JCM 32575^T^ is the type strain of *M. hoshinonis*. The optimum growth temperature for this strain is 15°C, and the maximum growth temperature is 20°C, but growth continues at near-zero and sub-zero temperatures. *M. hoshinonis* does not require amino acids and vitamins for growth and can grow even at −3°C (1). Here, we analyzed the draft genome sequence of this cryophilic yeast strain JCM 32575^T^.

The type strain of *Mrakia hoshinonis* is deposited at the Japan Collection of Microorganisms, Riken, Japan, as JCM 32575. The yeast culture and DNA extraction methods followed those previously reported (3–5). Briefly, yeast cells were cultured in the yeast extract–peptone–dextrose liquid medium (Difco) at 15°C for 7 days. The cells from cultures were collected by centrifugation at 3500 *g* for 7 minutes at 4°C. The cell pellets were washed with sterile distilled water and precipitated by centrifugation again. The cell pellets were dried by a vacuum freeze-dryer. The lyophilized cells were then powdered in a mortar and used for DNA extraction. The genomic DNA was extracted and purified using a NucleoBond AXG100 column with a Nucleo buffer set III (TaKaRa Bio) according to the manufacturer’s protocol. The concentration and purification of the genomic DNA were determined by NanoDrop (Thermo Scientific) and the Qubit dsDNA BR Assay Kits (Thermo Scientific). Then, the genomic DNA was digested using the microTUBE (Covaris), and the genomic library was constructed using the Illumina TruSeq DNA PCR-free library preparation kit (Illumina). The quality of the library was confirmed using the Quant-iT PicoGreen dsDNA assay kit, and the library size was checked using the Agilent 2200 TapeStation system (Agilent Technologies, Inc.). The paired-end sequence reaction was performed on a Novaseq 6000 instrument (Illumina) producing 250-bp sequence read lengths. Default parameters were used except where otherwise noted. Adapters and low-quality bases were trimmed using fastp ver. 0.12.4 (6) with the following parameter values: worker thread number was 4, the quality value was 20, and length_required was 41. Assembly of the nuclear genomes was conducted using Platanus ver. 1.2.4 (7). The completeness of the assemblies was assessed using BUSCO ver. 5.2.2 (8) with the database basidiomycota_odb10 (the number of the marker is 1764), after the identification of repeat sequences using RepeatModeler ver. 2.0.1 and RepeatMasker ver. 4.1.2 (9).

A summary of the genomic analysis results of the *M. hoshinonis* JCM 32575^T^ is shown in Table 1. This genomic information about *M. hoshinonis* offers insights into how fungi living in the High Arctic are adapted to the extreme polar environment.

**TABLE 1:** Assembly summary for draft genomic data of *Mrakia hoshinonis* JCM 32575^T^

Characteristic	JCM 32575^T^
Sampling site	Walker Glacier, Ellesmere Island
No. of reads	7,409,436,300
No. of scaffolds	266
Total length (Mb)	23.98
GC content (%)	59.9
Coverage (×)	304
Scaffold *N*_50_ value (kb)	353
BUSCO score completeness (%)	93.5
BUSCO score duplication (%)	0.5
GenBank accession No.	BTPH01000000.1
DRA accession No.	DRA016938.

## Data Availability

This Whole Genome Shotgun project has been deposited at DDBJ/EMBL/GenBank under BioProject No. PRJDB16469 with accession number BTPH01000000.1. The raw reads are available under DRA accession number DRA016938.
